# Simulation of High-Resolution Magnetic Resonance Images on the IBM Blue Gene/L Supercomputer Using SIMRI

**DOI:** 10.1155/2011/305968

**Published:** 2011-04-10

**Authors:** K. G. Baum, G. Menezes, M. Helguera

**Affiliations:** Chester F. Carlson Center for Imaging Science, Rochester Institute of Technology, 54 Lomb Memorial Drive, Rochester, NY 14623, USA

## Abstract

Medical imaging system simulators are tools that provide a means to evaluate system architecture and create artificial image sets that are appropriate for specific applications. We have modified SIMRI, a Bloch equation-based magnetic resonance image simulator, in order to successfully generate high-resolution 3D MR images of the Montreal brain phantom using Blue Gene/L systems. Results show that redistribution of the workload allows an anatomically accurate 256^3^ voxel spin-echo simulation in less than 5 hours when executed on an 8192-node partition of a Blue Gene/L system.

## 1. Introduction

The collection of medical image data for research can be an expensive and time-consuming task. Positron emission tomography (PET), X-ray computed tomography (CT), and magnetic resonance imaging (MRI) systems can easily cost over a million dollars. They may require dedicated staff, maintenance contracts, and access to expensive supporting equipment. In addition, collection of data for large clinical studies may take months. The process is complicated by equipment schedules, organization of volunteers/subjects, and involvement of potentially harmful electromagnetic radiation, radiopharmaceuticals, and contrast agents, as well as patient privacy rights. These difficulties limit the availability of clinical data, especially for smaller academic research programs.

Creating software models of the human anatomy and imaging systems and modeling the medical physics of the imaging acquisition process can provide a means to generate realistic synthetic data sets. In many cases, synthetic data sets can be used, reducing the time and cost of collecting real images and making data sets available to institutions without clinical imaging systems.

Synthetic data sets can be used for training purposes and as evaluation data for image processing and analysis algorithms. One additional advantage of synthetic data sets, that can make them an invaluable tool, is that they have a known ground truth. Ground truth refers to having exact knowledge of the object being imaged. Ground truth is, in many cases, nearly impossible to obtain for real images of living humans. In addition, system models can be used to improve system design and study imaging parameter selection and acquisition protocols.

While medical image simulation software has been under development since the 1980s, until recently the complexity of the procedures and long computation times have limited the realism and accuracy of artificially generated images. Improvements in computational systems have facilitated simulations that were previously infeasible. Advancements in processor architecture, increases in speed and amount of memory, and development of large storage systems have enabled computers to be used for increasingly complex problems. The use of distributed systems and technologies provides unparalleled computational capabilities.

With these technological improvements and an increased understanding of human anatomy and medical physics, three-dimensional high-resolution realistic synthetic medical data sets can be generated. In addition, the large quantity of images often necessary for studies, hundreds or thousands of images, can be quickly generated and made available. In some cases, simulations can even be performed in real time.

The remainder of this paper is dedicated to the generation of MR image sets. MR images are generally the most computationally complex images to generate, with simulations usually being performed in 2D or low-resolution 3D. What follows is, at least to the authors' knowledge, an overview of the first system capable of successfully generating high-resolution 3D MRI images.

## 2. Background

### 2.1. MRI Simulators

Simulators can be classified according to the procedure used to estimate the radio frequency response of the phantom (digital anatomy being imaged). In general, existing simulators fall into one of three categories: signal equation-based simulators [1, 2], *k*-space-based simulators [3], and isochromat summation-based simulators [4–10].

Signal equation-based simulators are the most common. Synthetic images are created using known tissue properties (i.e., spin density, spin lattice relaxation time, and spin-spin relaxation time) and the signal intensity equation for the pulse sequence being simulated. By substituting the known tissue properties into the signal equation, the simulated image can be calculated. For example, (1) is the signal equation for the standard spin-echo pulse sequence where *ρ* is the spin density, *T*_1_ is the spin lattice relaxation time, *T*_2_ is the spin-spin relaxation time, and TE and TR are the pulse sequence parameters representing the echo time and repetition time, respectively

(1) $$S=\rho \left(1-e^{-\text{TR}/T_{1}}\right)e^{-\text{TE}/T_{2}}.$$

Since the simulation is a simple mathematical calculation, simulated images can be created nearly instantaneously. Simulation is however limited to pulse sequences with a known signal equation, and generally more complex phenomena and artifacts cannot easily be simulated using this method.

A second, rarely used, approach to generate synthetic data sets leverages the *k*-space representation of the data. This simulation begins by approximating *k*-space by taking the Fourier transform of the phantom being imaged. This represents the ideal signal acquired by the scanner. The signal is then adjusted for each pulse event, for relaxation, and for the artifacts being simulated. While this technique provides a relatively computationally efficient way to obtain the MRI signal, it is difficult to simulate certain effects, such as field inhomogeneities. In addition, an understanding of how each event and artifact manifests itself in *k*-space is necessary requiring expert knowledge and making simulation of more complex artifacts difficult.

The third simulator type, and by far the most robust, arrives at the MRI signal by summing a large number of isochromats. The phantom is treated as a set of spin packets, and the evolution of the magnetization of each packet is governed by the Bloch equations [11]. A group of spins located in close proximity is represented as an isochromat. The rotation of the magnetization due to radio-frequency (RF) pulses, gradients, and relaxation is modeled. The final *k*-space signal is generated by summing the signal provided by each isochromat (isochromat summation), and the image is obtained by Fourier transforming the *k*-space data. These simulators are robust and in theory can simulate any pulse sequence and most phenomena. Limitations exist only due to the assumptions made by the Bloch equations and by computation time. This work attempts to ameliorate the situation by lessening the limitations imposed by computational complexity.

### 2.2. Simulation by Summation of Isochromats

Each isochromat is assigned a location within the phantom and is represented by a three-dimensional magnetization vector ([*M*_*x*_(*t*) *M*_*y*_(*t*) *M*_*z*_(*t*)]^*T*^), which specifies the isochromat's orientation and magnitude at time *t*. The location of the isochromat within the magnet bore is used to identify the magnetic field strength at the isochromat (due to the main static magnetic field, gradients, and any inhomogeneities such as from susceptibility). The local magnetic field strength (*B*) and the gyromagnetic ratio (*γ*) of the isochromat are used to find the frequency at which the isochromat precesses within the main magnetic field (Larmor frequency, *ν*) as follows:

(2) ν=γB.

After a period of time (Δ*t*) has passed, the Larmor frequency can be used to find the new orientation of the isochromat by modeling the precession as a rotation about the main field (usually oriented along the *z*-axis). This rotation is modeled as follows:

(3) $$\left[\begin{array}{c} M_{x}\left(t+\Delta t\right)\\ M_{y}\left(t+\Delta t\right)\\ M_{z}\left(t+\Delta t\right) \end{array}\right]=\left[\begin{array}{ccc} \cos\;\;\left(\nu \cdot \Delta t\right) & \sin\;\left(\nu \cdot \Delta t\right) & 0\\ -\sin\;\left(\nu \cdot \Delta t\right) & \cos\;\;\left(\nu \cdot \Delta t\right) & 0\\ 0 & 0 & 1 \end{array}\right]\left[\begin{array}{c} M_{x}\left(t\right)\\ M_{y}\left(t\right)\\ M_{z}\left(t\right) \end{array}\right].$$

In addition to precession within the main magnetic field, the magnitude of the isochromat changes due to relaxation effects. The transverse spin-spin relaxation is a result of molecular interactions causing a dephasing of the spins and a loss of signal coherence. The rate of relaxation is governed by the tissue-specific spin-spin relaxation time constant (*T*_2_) and affects the magnitude of the magnetization vector orthogonal to the main magnetic field. The effect of this dephasing on the magnetization vector can be modeled with

(4) $$\left[\begin{array}{c} M_{x}\left(t+\Delta t\right)\\ M_{y}\left(t+\Delta t\right)\\ M_{z}\left(t+\Delta t\right) \end{array}\right]=\left[\begin{array}{ccc} e^{-\Delta t/T_{2}} & 0 & 0\\ 0 & e^{-\Delta t/T_{2}} & 0\\ 0 & 0 & 1 \end{array}\right]\left[\begin{array}{c} M_{x}\left(t\right)\\ M_{y}\left(t\right)\\ M_{z}\left(t\right) \end{array}\right].$$

Longitudinal spin lattice relaxation occurs as the isochromats return to equilibrium (realign with the static magnetic field). Longitudinal relaxation is governed by the spin lattice relaxation time constant  (*T*_1_) and affects the magnitude of the magnetization vector in the direction of the main magnetic field, as calculated from

(5) $$M_{z}\left(t+\Delta t\right)=M_{z}\left(t\right)e^{-\Delta t/T_{1}}+\;M_{0}\left(1-e^{-\Delta t/T_{1}}\right).$$

Here, the magnitude of the isochromat's magnetization at equilibrium within the main magnetic field is given by *M*_0_. This magnitude is proportional to the isochromat's spin density.

Applying both relaxation effects simultaneously yields

(6) $$\begin{array}{cc} \left[\begin{array}{c} M_{x}\left(t+\Delta t\right)\\ M_{y}\left(t+\Delta t\right)\\ M_{z}\left(t+\Delta t\right) \end{array}\right] & =\left[\begin{array}{ccc} e^{-\Delta t/T_{2}} & 0 & 0\\ 0 & e^{-\Delta t/T_{2}} & 0\\ 0 & 0 & e^{-\Delta t/T_{1}} \end{array}\right]\left[\begin{array}{c} M_{x}\left(t\right)\\ M_{y}\left(t\right)\\ M_{z}\left(t\right) \end{array}\right]\\ & \;+\left[\begin{array}{c} 0\\ 0\\ M_{0}\left(1-e^{-\Delta t/T_{1}}\right) \end{array}\right]. \end{array}$$

Including the precession within the main static field yields (7) used to iteratively update the isochromat magnetization vector

(7) $$\begin{array}{cc} \left[\begin{array}{c} M_{x}\left(t+\Delta t\right)\\ M_{y}\left(t+\Delta t\right)\\ M_{z}\left(t+\Delta t\right) \end{array}\right] & =\left[\begin{array}{ccc} e^{-\Delta t/T_{2}} & 0 & 0\\ 0 & e^{-\Delta t/T_{2}} & 0\\ 0 & 0 & e^{-\Delta t/T_{1}} \end{array}\right]\\ & \;\times \left[\begin{array}{ccc} \cos\;\;\left(\nu \cdot \Delta t\right) & \sin\;\left(\nu \cdot \Delta t\right) & 0\\ -\sin\;\left(\nu \cdot \Delta t\right) & \cos\;\;\left(\nu \cdot \Delta t\right) & 0\\ 0 & 0 & 1 \end{array}\right]\left[\begin{array}{c} M_{x}\left(t\right)\\ M_{y}\left(t\right)\\ M_{z}\left(t\right) \end{array}\right]\\ & \;+\left[\begin{array}{c} 0\\ 0\\ M_{0}\left(1-e^{-\Delta t/T_{1}}\right) \end{array}\right]. \end{array}$$

**Figure 1:**
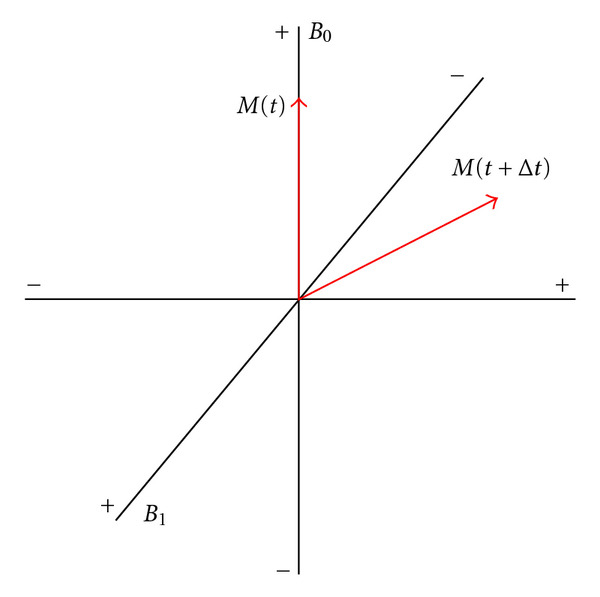
Application of a pulsed magnetic field (*B*_1_) orthogonal to the main static magnetic field (*B*_0_) results in the net magnetization of the isochromat rotating about the direction of *B*_1_.

RF pulses are used to excite the isochromats causing a deviation of their magnetic moments from equilibrium. An alternating current is run through a coil placed around the *x*-axis. Turning on and off the current at the Larmor frequency results in a pulsed magnetic field (*B*_1_) orthogonal to the main static magnetic field (*B*_0_). If we consider a frame of reference which rotates about the *z*-axis at the Lamor frequency, *B*_1_ will be a constant field along the *x*-axis. In this rotating frame of reference, the energy from the pulsed field will be absorbed by the isochromats causing them to rotate about the direction of the *B*_1_ field. This process is depicted in Figure 1. In the nonrotating (laboratory) frame of reference, the isochromats will follow a helical path making increasingly larger circles down towards the *xy*-plane.

We will now examine the effects of a rectangular pulse. The frequency of the pulse is selected, such that it targets specific isochromats. The local magnetic field strength (*B*_*t*_) and the gyromagnetic ratio (*γ*_*t*_) of the targeted isochromat are used to determine the frequency of the pulse (*ν*_*p*_) according to

(8) $$\nu_{p}=\gamma_{t}B_{t}.$$

The amount of rotation is dependent on the pulse duration and the strength of the *B*_1_ field. Isochromats precessing at *ν*_*p*_ will experience a rotation of *θ*_*t*_ degrees when a pulse with a magnitude of *B*_1_ is applied for a duration of *τ*, as given by (9).

(9) $$\theta_{t}=\gamma_{t}B_{1}\tau .$$

In reality, the rectangular pulse will contain other frequencies as well. Isochromats precessing at other frequencies may still absorb some of the energy causing them to experience smaller perturbations. The observed rotation will depend on the deviation of the isochromat's magnetic field strength (*B*_*i*_) from *B*_*t*_ and occur around the direction of the effective magnetic field *B*_1_′.

*B*_1_′ deviates from *B*_1_ by an angle *β*_*i*_, found using

(10) $$\beta_{i}=\tan^{-1}\;\left(\frac{B_{t}-B_{i}}{B_{1}}\right).$$

The angle of rotation will depend on the pulse duration and strength of the *B*_1_′ magnetic field, as given by

(11) $$\theta_{i}=\gamma_{i}\left|B_{1}^{\prime }\right|\tau =\gamma_{i}\tau \sqrt{{\left(B_{t}-B_{i}\right)}^{2}+{\left(B_{1}\right)}^{2}}.$$

This procedure is most easily implemented by applying a series of three rotations. First, a rotation of *β*_*i*_ around the *y*-axis aligns *B*_1_′ with the *x*-axis. The excitation by *θ*_*i*_ degrees can then be implemented by a rotation about the *x*-axis. Finally, a rotation around the *y*-axis of −*β*_*i*_ will recover the proper orientation. The process is represented mathematically by

(12) $$\begin{array}{cc} \left[\begin{array}{c} M_{x}\left(t+\tau \right)\\ M_{y}\left(t+\tau \right)\\ M_{z}\left(t+\tau \right) \end{array}\right] & =\left[\begin{array}{ccc} \cos\;\;\beta_{i} & 0 & -\sin\;\beta_{i}\\ 0 & 1 & 0\\ \sin\;\beta_{i} & 0 & \cos\;\;\beta_{i} \end{array}\right]\left[\begin{array}{ccc} 1 & 0 & 0\\ 0 & \cos\;\;\beta_{i} & \sin\;\beta_{i}\\ 0 & -\sin\;\beta_{i} & \cos\;\;\beta_{i} \end{array}\right]\\ & \;\times \left[\begin{array}{ccc} \cos\;\;{-\beta }_{i} & 0 & -\sin\;{-\beta }_{i}\\ 0 & 1 & 0\\ \sin\;{-\beta }_{i} & 0 & \cos\;\;{-\beta }_{i} \end{array}\right]\left[\begin{array}{c} M_{x}\left(t\right)\\ M_{y}\left(t\right)\\ M_{z}\left(t\right) \end{array}\right]. \end{array}$$

More complex pulses, such as sin (*x*)/*x*, can be represented as a series of short rectangular pulses.

Generally, a restriction is placed that *τ* is much smaller than *T*_1_ and *T*_2_, so that no relaxation or dephasing occurs as the pulse is being applied. In this case, the final result of applying the pulse in the rotating frame of reference is equivalent to applying the pulse in the laboratory frame.

As previously mentioned, each isochromat is under the influence of the local magnetic field (*B*) present at its spatial location within the scanner. This field determines the precessing frequency of the isochromat as well as its behavior during the application of an RF pulse. The local magnetic field strength is the sum of the main static magnetic field (*B*_0_), along with any variation due to tissue susceptibility, any inhomogeneities in the main field that have not been corrected by proper shimming, and any gradients that are currently being applied. Gradients are applied linearly across the *x*, *y*, and *z*-axes of the system. The change in local magnetic field strength due to the gradients (Δ*B*_*G*_) can be calculated with (13), where (*x*, *y*, *z*) is the spatial location of the isochromat, and *G*_*x*_, *G*_*y*_, and *G*_*z*_ are the strengths of the applied gradients along the *x*, *y*, and *z*-axes of the system, respectively

(13) $${\Delta B}_{G}=xG_{x}+\;yG_{y}+zG_{z}.$$

Additional local modifications can be made for imperfections in the gradient. The gradients applied during image acquisition are defined by the pulse sequence and serve purposes such as phase and frequency encoding and slice selection. They can be applied anytime during acquisition including during precession, during the application of a pulse, or during signal acquisition.

Signal acquisition is typically achieved by the placement of two orthogonal coils. Precession of the isochromats induces an observable current in the coils. This signal is typically represented in complex notation where the real component is the signal observed by the first coil, and the imaginary component is the signal observed by the second coil.

The observed signal is calculated by summing the magnetization of all the isochromats along the direction of observation. For example, if the coils are placed along the *x*- and *y*-axes, the real component can be the sum of the magnetization along the *x*-axis, and the imaginary component can be the sum of the magnetization along the *y*-axis.

The observed signal consists of multiple samples collected over a short period of time which will define one line of *k*-space. This is accomplished by iteratively acquiring a sample and performing a precession.

A typical acquisition process will consist of repeatedly applying a pulse sequence (with varying parameters such as the phase-encoding gradient strength) and collecting signal samples to fill a line of *k*-space. This will be repeated until samples have been collected for all of *k*-space. An image reconstruction algorithm is then applied to transform the *k*-space data into an image.

One area in which isochromat simulators vary is in the way they accurately simulate the spin-echo phenomenon [12]. Echos occur when spins that were previously dephased come back into alignment. Accurate echo simulation requires the use of a large number of isochromats per voxel of the reconstructed image, greatly increasing execution time. Shkarin and Spencer provide a nice overview of this issue [7]. Alternative methods have been proposed including using a time variable to track when rephasing should occur [8], using gradient magnitudes to predict the amount of dephasing that occurs in the region represented by an isochromat [6], and calculating intravoxel magnetization gradients [13].

## 3. Materials and Methods

### 3.1. SIMRI

**Figure 2:**
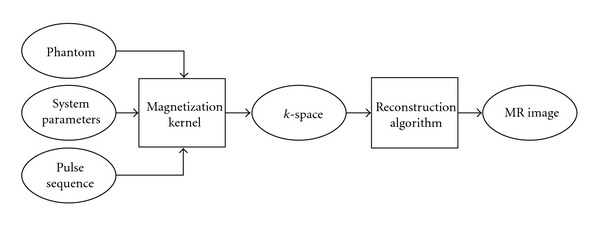
Overview of the process that SIMRI uses to create synthetic MR images.

SIMRI is a Bloch equation-based magnetic resonance image simulator, developed in CREATIS, Lyons, France [8]. The source code is downloadable [14]. It includes a few pulse sequences, and the framework allows for the implementation of ad hoc ones. Given a digital phantom of an object, the magnetic resonance signal can be generated if the fractional tissue components of each voxel are known. It supports static field inhomogeneities due to improper shimming and tissue susceptibility, features efficient modeling of intravoxel inhomogeneities, and properly models the main artifacts such as susceptibility, wrap around, chemical shift, and partial volume effects. The design of SIMRI is shown in Figure 2. SIMRI is designed to take advantage of small clusters supporting the message passing interface (MPI) communications protocol, but modifications are necessary to take advantage of larger parallel computing solutions.

### 3.2. Montreal Brain Phantom

Research at McGill University has resulted in a set of high-resolution voxel-based brain phantoms [15–17]. These phantoms were created by semiautomatically segmenting real brain data sets. In order to accurately identify all of the tissues of interest, the segmentation was based on *T*_1_, *T*_2_, and proton density weighted magnetic resonance images, magnetic resonance angiography (MRA), and computed tomography (CT) images. The resulting high-resolution phantom consists of 11 tissue types: grey matter, white matter, cerebrospinal fluid, skull, marrow within the bone, dura, fat, tissue around the fat, muscles, skin/muscles, and vessels. The phantom is presented as a set of fuzzy volumes, one volume for each tissue type, where the voxel intensities represent the amount of that tissue in the given voxel.

### 3.3. Blue Gene

Blue Gene is a massively parallel computer developed by IBM in collaboration with several partners including the Lawrence Livermore National Laboratory. For this work, two Blue Gene/L systems available in New York were used. New York Blue is the system hosted at Brookhaven National Laboratory consisting of 18,432 dual 700 MHz PowerPC 440 nodes with 1024 MB of memory. Rensselaer's Computational Center for Nanotechnology hosts another system consisting of 16,384 dual 700 MHz PowerPC 440 nodes divided equally between 512 MB and 1024 MB configurations.

Blue Gene/L utilizes system-on-a-chip technology, integrating all functionality of a node, except for main memory, into a single integrated circuit. The use of lower clock speed processors in addition to this high level of integration allows a highly scalable architecture at low cost and low power consumption [18].

Compute nodes consist of two double floating point core processors, supporting an enhanced instruction set including single instruction and multiple data (SIMD) operations. They come in 512 MB and 1 GB SDRAM configurations. Two operation modes are provided: communication coprocessor mode in which one CPU is used for computation and the other for communication and virtual node mode in which both CPU are leveraged for computation. In virtual node mode, the memory is divided between the two cores. Nodes are connected into a 3D torus, with the smallest unit being 4 × 4 × 2 [18].

Dedicated I/O nodes with Gigabit Ethernet provide an interface between the compute nodes and external systems including the file systems. I/O node to compute node ratios of 1 : 8 to 1 : 256 is supported [18].

Applications built on the Blue Gene/L system utilize message passing interface (MPI) for communication between nodes. Compute nodes run a customized light-weight Linux kernel. There are significant differences between a Blue Gene/L compute node execution environment and a standard personal workstation execution environment [19].

The most notable difference is that Blue Gene/L nodes do not support virtual paging. This means that unlike the 2 or 3 GB of virtual memory typically available to any processes running on a 32-bit system, regardless of physical memory amounts, a Blue Gene/L node is limited by the actual physical memory. The application, kernel, and MPI communication buffers must total less than the 256 MB, 512 MB, or 1024 MB of physical memory available to the node. This requires applications to have a very low memory footprint per node, carefully manage communication buffers, and be clean of any memory leaks that can quickly consume the available memory [19].

### 3.4. Code Modifications

Several modifications needed to be made in order for SIMRI to run successfully on the Blue Gene/L systems. The modified version of SIMRI can be requested by contacting the corresponding author. The most significant modifications included redistribution of the workload and optimization of the memory.

Since each isochromat progresses independently, parallelization is straightforward. Essentially a set of isochromats can be assigned to each computation node. The progression of the isochromats can then be independently modeled and the resulting acquired signals summed together at the end. In SIMRI, this was done by assigning one slice (i.e., plane) of the phantom object to each node. The number of divisions (and participating compute nodes) is limited by the dimension of the image. This division of labor is appropriate for small clusters, but not for massively parallel systems like the Blue Gene/L. In order to leverage the additional compute nodes, a finer division of labor is needed. Modifications were made, so assignment of tasks no longer needs to occur around slice boundaries and that arbitrary groups of isochromats can be assigned to each node.

The simulation has been modified to proceed as follows. (1) When launching the application, a number of manager nodes are specified. (2) The remaining nodes are considered workers. Each worker is assigned to one of the manager nodes in a manner, such that each manager has the same number of workers. (3) The isochromats to be modeled are divided up evenly between the manager nodes. (4) Each manager node further divides up the isochromats amongst its workers. (5) The worker nodes perform the simulation and return back to their manager the MR signal from the isochromats they were responsible for modeling. (6) The manager nodes combine the signals from each of its workers. (7) The accumulated signal from each manager is then combined to create the final MR signal. (8) This MR signal can then be reconstructed to produce the simulated image. This tree-like distribution network allows efficient use of the large number of nodes in the Blue Gene/L systems.

Initial test runs required approximately 2.7 GB of memory per node for a 256^3^ voxel simulation. This is well in excess of the 1 GB maximum of the Blue Gene/L nodes. Several steps were taken which allowed this requirement to be met. The memory usage pattern was modified to retain memory only as long as necessary and to reuse previously allocated blocks. The MPI messaging schedule was modified to reduce the size of messages. This is important as the buffers internally allocated by MPI can consume considerable memory if not careful. Finally, the code executed on each node was made more specialized for the task the node would be performing. The increased specialization of each node results in memory being allocated conditionally based on the node's role. As a result of these modifications, the memory use is sufficiently below the 1 GB that is shared between the application and the kernel on the BlueGene/L systems.

## 4. Results

**Figure 3:**
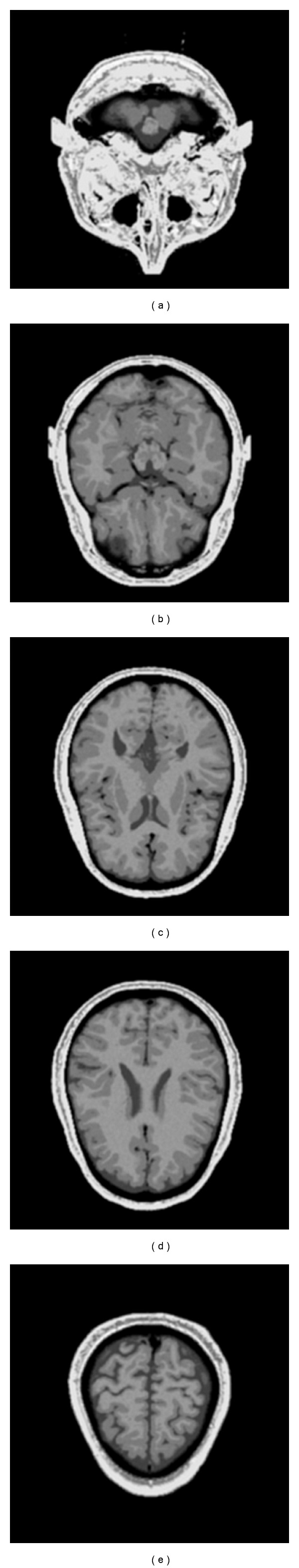
Selected slices from the 256^3^ voxel simulated image of the Montreal brain phantom.

Sample results from a 256^3^ voxel simulation are shown in Figure 3. This 256^3^ voxel spin-echo simulation took approximately 4.3 hours executing on 8192 nodes of the BlueGene/L system. A full 3D acquisition can easily and quickly be simulated.

**Figure 4:**
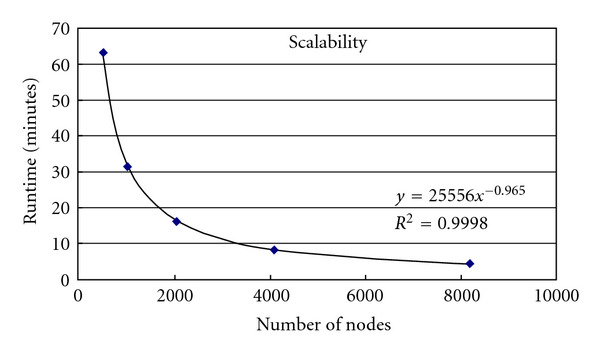
Scalability of the modified SIMRI running on the Blue Gene/L system. The scalability is nearly linear (1/0.965).

Benchmark runs were performed using the Montreal brain phantom and a spin-echo protocol. A 128^3^ voxel simulation was performed using 512, 1024, 2048, 4096, and 8192 nodes. Table 1 shows the run times for each simulation and the scalability (run time versus number of nodes) is plotted in Figure 4. As expected, the run time is found to be inversely proportional to the number of nodes used.

**Figure 5:**
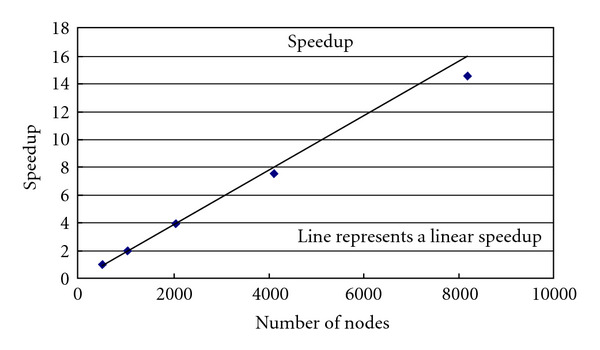
Speedup of the modified SIMRI running on the Blue Gene/L system. The plotted line represents a perfect linear speedup.

**Figure 6:**
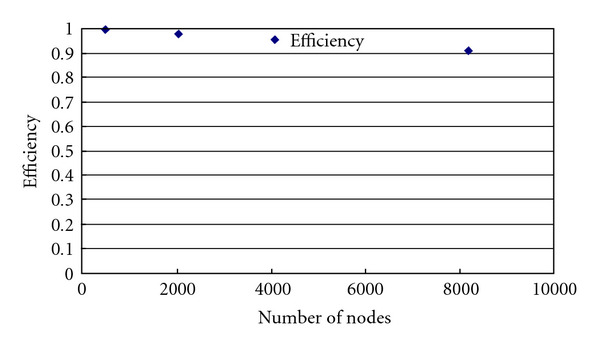
Efficiency of the modified SIMRI running on the Blue Gene/L system. The efficiency is near unity decreasing as the number of nodes approaches the number of isochromats and the communication/computation ratio increases.

This provides a nearly linear speedup (see Figure 5). The efficiency (speedup divided by number of nodes), plotted in Figure 6, is approximately unity tapering off only slightly as the number of nodes approaches the number of isochromats and the communication/computation ratio increases.

A set of simulations were run on a single workstation in order to emphasize the benefit of a distributed approach. A workstation equipped with an Intel Xeon X5260 (3.33 GHz) processor was used to run two-dimensional and three-dimensional spin-echo simulations of a phantom consisting of a single spherical object. The resulting run times are shown in Table 2. Using a single workstation in it is possible to perform two-dimensional and small three-dimensional simulations. Higher-resolution simulations may however require a prohibitive amount of time, and it is may not be feasible to create a large set of simulated data.

## 5. Conclusions

Modern distributed systems provide unparalleled computational capabilities allowing full resolution data sets to be generated in reasonable amounts of time. The growing availability of these systems, and the public funding for them, has resulted in them being readily available to a large number of academic researchers.

SIMRI has proved to be a robust modeling package that can easily be modified to target a variety of distributed computing environments. As shown here, it can be used to model the image acquisition process allowing the generation of realistic high-resolution images. In addition, large quantities of simulated images can be generated enabling their use in research studies (for example, see [20]). Collections of realistic simulated data sets can be used for validation of image processing algorithms, perception studies, and training.

Historically computational complexity has limited the accuracy of medical image simulations. As technology continues to evolve, the realism of the simulated data will be limited by the accuracy of the image acquisition process model and the accuracy of the anatomical model being imaged.

## Figures and Tables

**Table 1:** Runtimes for a 128^3^ simulation using different numbers of nodes.

Number of nodes	512	1024	2048	4096	8192
Runtime (min.)	63.2	31.4	16.1	8.3	4.4

**Table 2:** Execution times for running SIMRI on a single workstation equipped with an Intel Xeon X5260 (3.33 GHz) processor.

Simulation size	128^2^	256^2^	512^2^	1024^2^	32^3^	64^3^	128^3^
Runtime (min.)	0.8	13.2	222.1	3418.9	3.3	219.5	13,385
